# Ethical issues with brain-computer interfaces

**DOI:** 10.3389/fnsys.2014.00136

**Published:** 2014-07-30

**Authors:** Walter Glannon

**Affiliations:** Department of Philosophy, Faculty of Arts, University of CalgaryCalgary, AB, Canada

**Keywords:** benefit-risk ratios, brain-computer interface, communication, expectation, auditory feeedback, visual feedback

## Introduction

Brain-computer interfaces (BCIs), or brain-machine interfaces (BMIs) involve real-time direct connections between the brain and a computer (Kubler, 2009; Wolpaw and Wolpaw, 2011). Bidirectional feedback between the user and the system produces physical changes that can restore some degree of motor or communicative control for individuals with lost limbs, extensive paralysis or who are significantly neurologically compromised (Hochberg et al., 2006, 2012). In these respects, a BCI can enable an individual with severe brain or bodily injury to regain some degree of agency. By providing the subject with the relevant type of feedback, the device may enable her to translate an intention into an action despite the inability to perform voluntary bodily movements. There are two types of feedback with a BCI. The first concerns feedback about the outcome of a self-initiated, BCI-mediated action, such as moving a computer cursor or robotic arm. It provides only indirect feedback about brain activity. The second type concerns direct feedback about the level of brain activity itself. The first is more pertinent to the potential to restore some behavior control in the sense that one can perceive the success or failure of their mental act. Although it is still at an early stage of development, an EEG- or fMRI-based BCI might also enable minimally conscious individuals or those with complete locked-in syndrome to communicate wishes about medical treatment when they are unable to do this verbally or gesturally (Sellers, 2013). These applications of interface technology raise a number of ethical issues (McCullagh et al., 2014), three of which I will discuss in this article. First, in some cases patients' and caregivers' expectations about recovering motor function with a BCI might not be reasonable given the cognitive challenges in operating the system. This might result in psychological harm when the subject's desires and intentions to produce actions fail to be realized. Second, the different types of electrodes used to detect and respond to motor cortical neural signals involve different levels of invasiveness and different benefit-risk ratios that have to be weighed with a view to the probable success or failure of the technique. Third, the use of a BCI for communication in neurologically compromised patients prompts the question of whether their responses would be evidence of the capacity to make informed decisions about their care.

## Expectations

The user of a BCI can execute an intention to perform a motor task through changes in the system caused by electrodes detecting signals in, for instance, the motor cortex mediating the intention. Success in operating the system depends on a combination of unconscious operant conditioning of brain responses and conscious goal-directed expectation of the subject. These depend in turn on how effective the practitioner is in training the subject how to operate the system. As in other cases of traumatic brain injury, goal-directed thinking in some patients with tetraplegia may be impaired if there is significant damage to neural networks in frontal regions mediating planning and decision-making. This may also impair the subject's capacity to understand the benefits and risks of the technique and give informed consent to participate in BCI research and treatment (Hochberg and Cochrane, 2013).

Ordinarily, motor skills are performed unconsciously and automatically following an initial period of conscious attention and learning. For those with severe paralysis, however, sustained attention is required both while being trained to operate the interface and effectively operating it to execute motor tasks. Subjects whose cognitive capacity for planning has been impaired by injury to the central nervous system may have difficulty in translating their thoughts into actions or fail to do so. Failure to meet the expectation to produce certain actions may cause distress and harm in some subjects by defeating their interest in recovering some, albeit limited, degree of motor control. Planning is a critical component in moving a prosthetic limb, for example. The subject must indicate with his brain and mind where the limb should go before executing the intention to move it. The cognitive workload requires considerable time and effort. This may cause frustration and anxiety and increase the probability of failure for some in trying to achieve their goal. It can exacerbate the feeling of a loss of behavior control. To minimize the probability of harm, investigators and practitioners must educate users on the potential positive effects and limits of BCIs. They should also adopt strict selection criteria and include only those with largely preserved cognitive functions who could give informed consent and would more likely be trained to successfully operate it. This may seem unfair to those with impaired levels of cognition who lack these capacities. Nevertheless, the idea of providing equal opportunity for all paralyzed individuals to access to a BCI would have to be weighed against the potential for emotional harm if a subject cannot meet the cognitive demands of operating the system and his expectations are not met. Discriminating on the basis of levels of cognitive function may be justified on these grounds.

## Benefits and risks

BCIs utilize wired or wireless systems to detect and allow transmission of signals in the motor cortex into actions. The significance of these systems for benefit and risk to patients depends not so much on the type used but their level of invasiveness. Theoretically, the distinction between wired and wireless systems is orthogonal to this level. The non-invasive type consists of scalp-based electrodes that are part of the equipment required to record EEG. Because they do not involve intracranial surgery and implantation of a device in the brain, they do not involve a risk of infection or hemorrhage. At the same time, though, they may not readily read signals from the motor cortex because the cranium can smear them.

In electrocorticography (ECoG), electrodes are implanted epidurally or subdurally (Leuthardt et al., 2004). These can decode motor cortical signals more readily than scalp-based electrodes because they are not susceptible to cranial smearing. But they entail some risk of infection and hemorrhage. Like the non-invasive system, both forms of ECoG BCIs impose constraints on the subjects' freedom from the wires running from the electrodes to the machine. Wireless systems consisting of a microelectrode array implanted in the motor cortex avoid this problem and are less burdensome for subjects. Because they can decode and transmit signals from this region more directly, implanted arrays are more likely to facilitate the execution of the subject's intentions in actions. Still, this would depend on the specifics of the neurological deficit and the patient's ability to manipulate the BCI. Moreover, in addition to the risk of infection and hemorrhage, microelectrode arrays raise the issue of biocompatibility between the implanted objects and surrounding neural tissue. The electrodes may reorganize and induce changes in the tissue. These changes may be salutary, especially if they promote neuroplasticity and the generation of new neuronal connections that could bypass the site of brain or spinal cord injury causing loss of motor function. But they could also cause adverse changes in the surrounding tissue and result in neurological and psychological sequelae. A safe and effective array that could function for many years would be one in which the surrounding neuropil grew into the electrode. This would be more stable and allow myelated axons to be recorded using implanted amplifiers (Kennedy et al., 2011). If this occurs, then invasive systems can be functionally superior to and as safe as non-invasive systems. The first type can have a more favorable benefit-risk ratio than the second.

## Communicating with a BCI

EEG- and fMRI-based BCIs might enable individuals to reliably communicate when they are unable to communicate behaviorally (Birbaumer et al., 2008, 2014). This involves three distinct patient groups. Minimally conscious patients have residual awareness of self and surroundings. Locked-in patients are fully aware despite being almost completely paralyzed. Some of these patients can communicate through voluntary eyelid movements. These in turn are distinct from completely locked-in patients who lack the capacity for any voluntary bodily movements. Conscious perception and expression of intentions in locked-in patients is different from that of minimally conscious patients, and this may better facilitate communication through a BCI. One challenge for this intervention would be that BCIs typically utilize visual feedback, and minimally conscious and completely locked-in subjects have limited or no capacity to receive feedback from and respond to a visual stimulus in learning how to operate the system. Alternatively, tactile or auditory feedback could be used to enable communication (Kubler, 2009; Hochberg and Cudkowicz, 2014). Yet even if this modality could overcome the limitations associated with a lack of visual feedback, questions would remain about the meaning of “communicate.” Specifically, it is not clear whether the responses of linguistically impaired minimally conscious or even fully conscious locked-in patients would be evidence of the cognitive and emotional capacity to give informed consent to continue or discontinue artificial hydration and nutrition (Brady Wagner, 2003; Jox, 2013).

Some investigators have claimed that fMRI-guided BCIs could enable minimally conscious patients with a high level of cognitive function to make these decisions (Peterson et al., 2013). But emotionally laden decisions about life-sustaining treatment reflect a person's values and attitudes about quality of life. It is questionable whether these values and attitudes can be expressed by simple “Yes” or “No” responses to questions (Monti et al., 2010), and yet they have to be included in any robust sense of “communication.” This involves more than being aware, even fully aware. More sophisticated interface systems enabling the expression of complex semantic processing may or may not confirm that the patient had the requisite capacities. Hochberg and Cudkowicz point out that among completely locked-in patients there have been “no reports of restoring communication using a neural signal-based BCI in this most severely affected population” (Hochberg and Cudkowicz, 2014, p. 1852; Birbaumer et al., 2014). Moreover, Fernandez-Espejo and Owen acknowledge that, with current interface technology, simple affirmative or negative responses to questions about whether a minimally conscious patient wanted to continue living would not be sufficient to establish that the patient had the “cognitive and emotional capacity to make such a complex decision” (Fernandez-Espejo and Owen, 2013, p. 808). But they also say that “it is only a matter of time before all of these obstacles are overcome” (p. 808).

This last point may be overly optimistic. Even advanced BCIs that could detect neural activity correlating with complex semantic processing might not be sufficient to show that the subject had the cognitive and emotional capacity to make an informed and autonomous decision about life-sustaining treatment. Some form of behavioral interaction may be necessary to confirm that the subject had this capacity. Medical professionals and caregivers must be cautious not to read too much into BCI-enabled responses and interpret them as having a meaning they lack.

## Conclusion

BCIs can benefit individuals by restoring varying degrees of motor control and possibly the ability to communicate. But expectations of some subjects and their caregivers may exceed what they can reasonably achieve with the technology and result in psychological harm. Selecting candidates with higher levels of preserved cognitive function for BCI research and treatment and educating them on the potential benefits and limitations of the technique is advisable to prevent or at least minimize harm. The fact that a particular BCI system is more invasive than others does not imply that it has an unacceptable degree of risk and may instead indicate a more favorable benefit-risk ratio if it does more to enable the execution of intentions in actions and promote neuroplasticity. Perhaps the most significant application of BCIs would be in enabling minimally conscious or completely locked-in patients to communicate. Yet it is questionable that even the most sophisticated system alone could demonstrate that these subjects have the ability to clearly and meaningfully communicate their wishes and make informed decisions about life-sustaining treatment. Decision-making capacity falls along a continuum correlating with a continuum of cognitive and emotional capacities, and there is no algorithm providing a definitive answer to the question of where the threshold at which one has a sufficient degree of these capacities lies. All relevant parties need to be cautious and not infer that a BCI indicating certain levels of brain activity and semantic processing in a subject is evidence of an understanding of the ethical magnitude of life-and-death decisions and the ability to make them.

### Conflict of interest statement

The author declares that the research was conducted in the absence of any commercial or financial relationships that could be construed as a potential conflict of interest.

## References

[B1] Birbaumer N. Gallegos-Ayala G. Wildgruber M. Silvoni S. Soekada S. (2014). Direct brain control and communication in paralysis. Brain Topogr. 27, 4–11 10.1007/s10548-013-0282-1 23536247

[B2] Birbaumer N. Murguialday A. Cohen L. (2008). Brain computer interface in paralysis. Curr. Opin. Neurol. 21, 634–638 10.1097/WCO.0b013e328315ee2d 18989104

[B3] Brady Wagner L. (2003). Clinical ethics in the context of language and cognitive impairment: rights and protections. Semin. Speech Lang. 24, 275–284 10.1055/s-2004-815581 14722801

[B4] Fernandez-Espejo D. Owen A. (2013). Detecting awareness after severe brain injury. Nat. Rev. Neurosci. 14, 801–809 10.1038/nrn3608 24088810

[B5] Hochberg L. Bacher D. Jarosiewicz B. Masse N. Simeral J. Vogel J. (2012). Reach and grasp by people with tetraplegia using a neurally controlled robotic arm. Nature 485, 372–375 10.1038/nature11076 22596161 PMC3640850

[B6] Hochberg L. Cochrane T. (2013). Implanted neural interfaces: ethics in treatment and research, in Neuroethics in Practice, eds Chatterjee A. Farah M. (New York, NY: Oxford University Press), 235–250 10.1093/acprof:oso/9780195389784.003.0017

[B7] Hochberg L. Cudkowicz M. (2014). Locked in, but not out? Neurology 82, 1852–1853 10.1212/WNL.0000000000000460 24789868

[B8] Hochberg L. Serruya M. Friehs G. Mukand J. Saleh M. Caplan A. (2006). Neuronal ensemble control of prosthetic devices by a human with tetraplegia. Nature 442, 164–171 10.1038/nature04970 16838014

[B9] Jox R. (2013). Interface cannot replace interlocution: why the reductionist concept of neuroimaging-based capacity determination fails. AJOB Neurosci. 4, 15–17 10.1080/21507740.2013.827279

[B10] Kennedy P. Andreasen D. Bartels J. Ehirm P. Mao H. Velliste M. (2011). Making the lifetime connection between brain and machine for restoring and enhancing function. Prog. Brain Res. 194, 1–25 10.1016/B978-0-444-53815-4.00020-0 21867791 PMC4305334

[B11] Kubler A. (2009). Brain-computer interfaces for communication in paralysed patients and implications for disorders of consciousness, in The Neurology of Consciousness: Cognitive Neuroscience and Neuropathology, eds Laureys S. Tononi G. (Amsterdam: Elsevier), 217–233 10.1016/B978-0-12-374168-4.00017-4

[B12] Leuthardt E. Schalk G. Wolpaw J. Ojemann J. Moran D. (2004). A brain-computer interface using electrocorticographic signals in humans. J. Neural Eng. 1, 63–71 10.1088/1741-2560/1/2/001 15876624

[B13] McCullagh P. Lightbody G. Zygierewicz J. Kernohan W. (2014). Ethical challenges associated with the development and deployment of brain computer interface technology. Neuroethics 7, 109–122 10.1007/s12152-013-9188-6

[B14] Monti M. Vanhaudenhuyse A. Coleman M. Boly M. Pickard J. Tshibanda L. (2010). Willful modulation of brain activity in disorders of consciousness. N. Engl. J. Med. 362, 579–598 10.1056/NEJMoa0905370 20130250

[B15] Peterson A. Naci L. Weijer C. Cruse D. Fernandez-Espejo D. Graham M. (2013). Assessing decision-making capacity in the behaviorally nonresponsive patient with residual covert awareness. AJOB Neurosci. 4, 3–14 10.1080/21507740.2013.821189

[B16] Sellers E. (2013). New horizons in brain-computer interface research. Clin. Neurophysiol. 124, 2–4 10.1016/j.clinph.2012.07.012 22902247 PMC3658460

[B17] Wolpaw J. Wolpaw E. (2011). Brain-Computer Interfaces: Principles and Practice. New York, NY: Oxford University Press

